# Accuracy of aortic root annulus assessment with cardiac magnetic resonance in patients referred for transcatheter aortic valve implantation: a comparison with multi-detector computed tomography

**DOI:** 10.1186/1532-429X-15-S1-O12

**Published:** 2013-01-30

**Authors:** Gianluca Pontone, Daniele Andreini, Erika Bertella, Saima Mushtaq, Sarah Cortinovis, Andrea Annoni, Alberto Formenti, Giovanni Ballerini, Mauro Pepi

**Affiliations:** 1 Centro Cardiologico Monzino IRCCS, Milan, Italy

## Background

To compare the accuracy of cardiac magnetic resonance (CMR) evaluation of the aortic root as compared to multi-detector computed tomography (MDCT) in patients referred for transcatheter aortic valve implantation (TAVI).

## Methods

In 50 patients, the following parameters were assessed with CMR and compared with those obtained with MDCT: aortic annulus (AoA) maximum diameter (AoA-Dmax), minimum diameter (AoA-Dmin), and area (AoA-A), length of the left coronary, right coronary, and non-coronary aortic leaflets, degree (grades 1 to 4) of aortic leaflet calcification and distance between AoA and coronary artery ostia.

## Results

AoA-Dmax, AoA-Dmin and AoA-A were 26.45±2.83 mm, 20.17±2.20 mm, 444.88±84.61 mm2 and 26.45±2.76 mm, 20.59±2.35 mm and 449.78±86.22 mm2 by MDCT and CMR, respectively. The length of left coronary, right coronary, and non-coronary leaflets were 14.02±2.27 mm, 13.33±2.33 mm, 13.39±1.97 mm, and 13.95±2.18 mm, 13.30±2.14 mm, 13.46±1.80 mm by MDCT and CMR, respectively, while the scores of aortic leaflet calcifications were 3.4±0.7 vs. 2.97±0.77. Finally, the distance between AoA and left main and right coronary artery ostia was 16.21±3.07 mm, 16.02±4.29 mm and 16.14±2.83 mm, 16.14±4.36 mm by CCT and CMR, respectively. There was close agreement between CMR and MDCT measurements, whereas aortic leaflet calcifications were underestimated by CMR.

## Conclusions

Aortic root assessment with CMR including AoA size, aortic leaflet length and coronary artery ostia height is accurate in comparison to MDCT. CMR may be a valid imaging alternative in patients unsuitable for MDCT.

## Funding

None.

